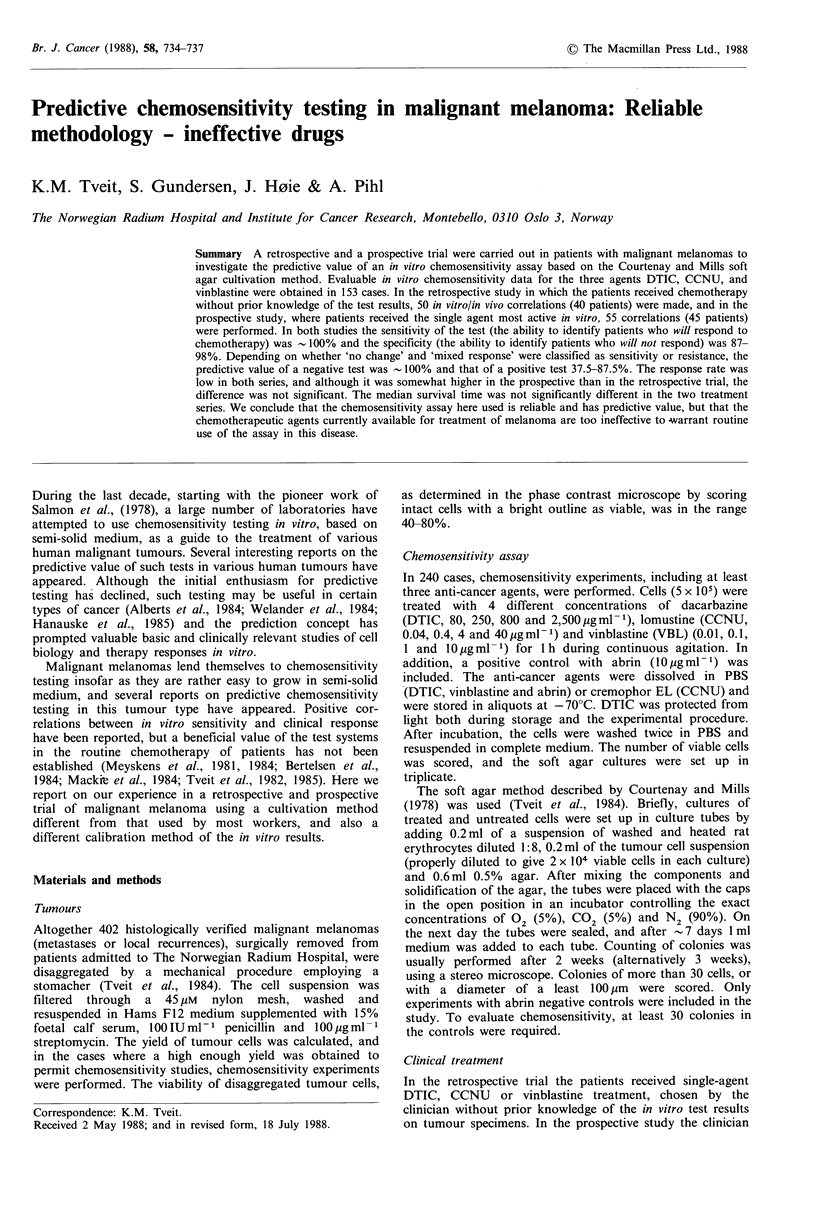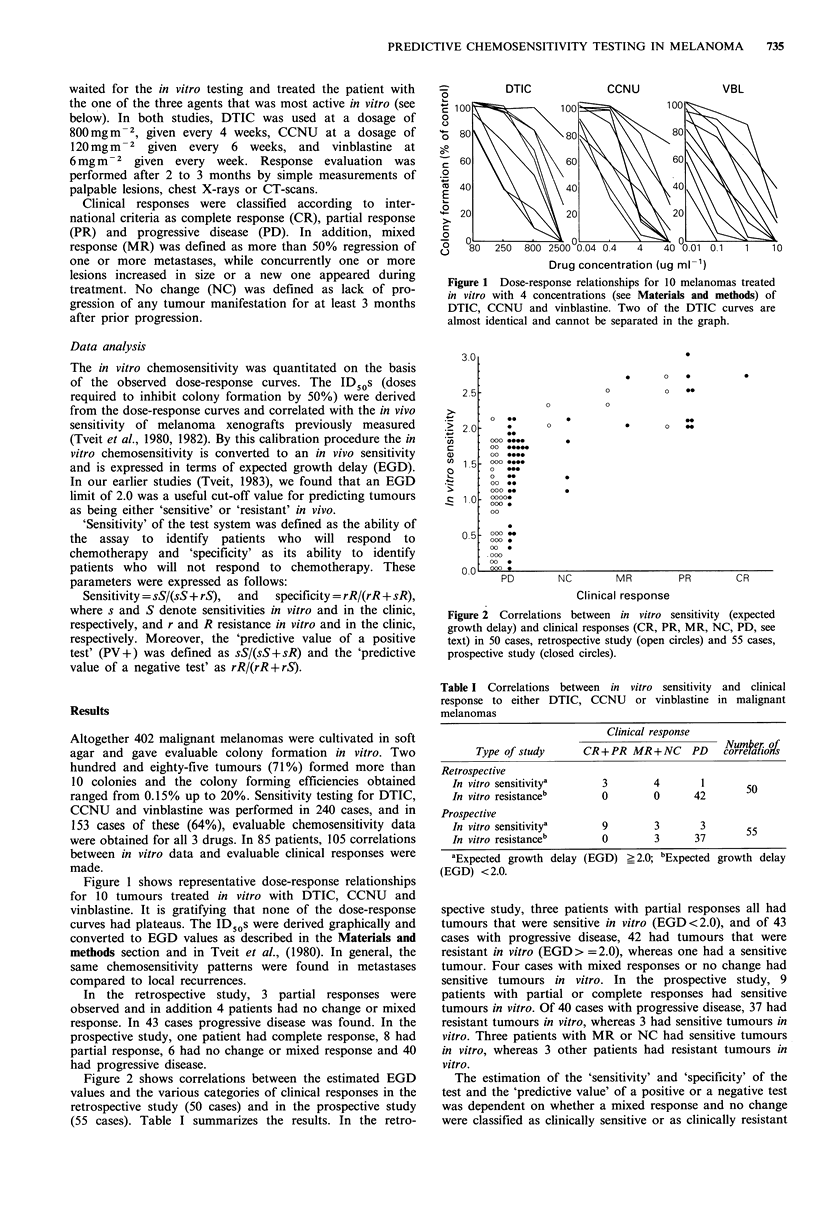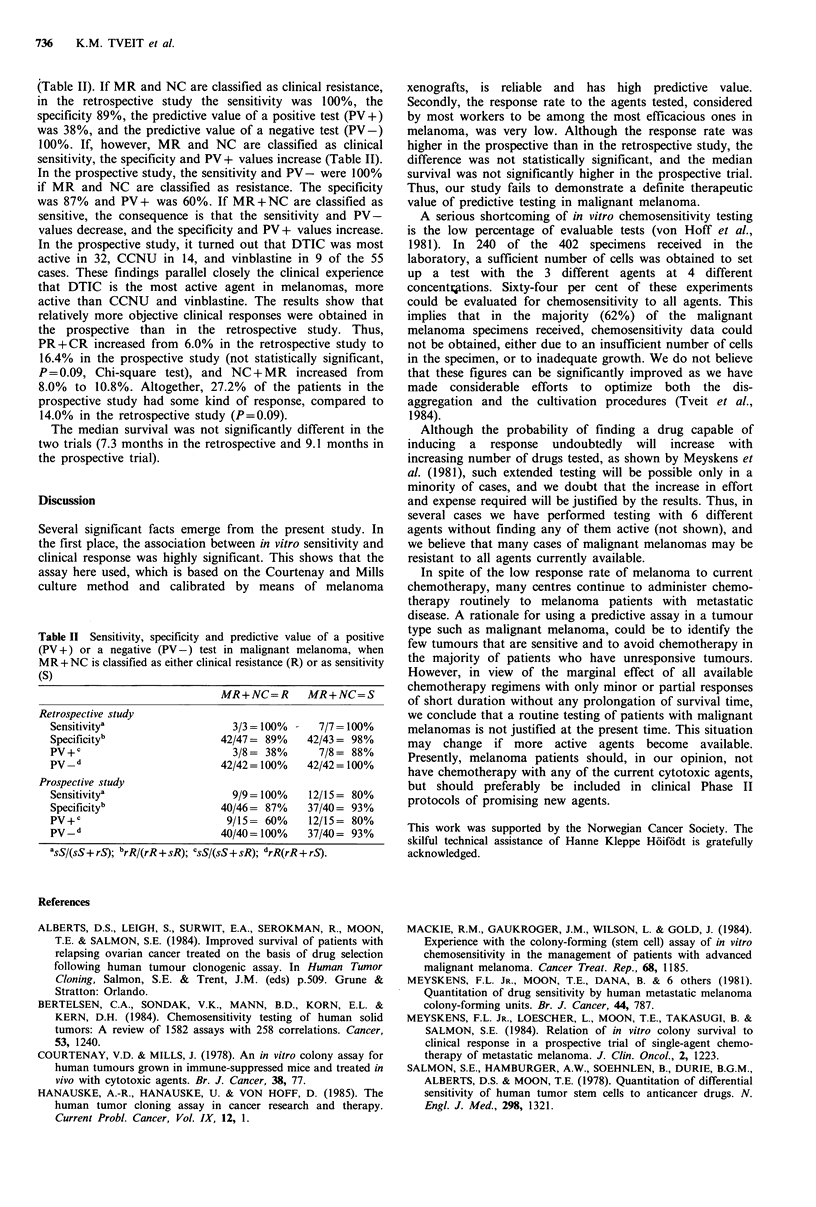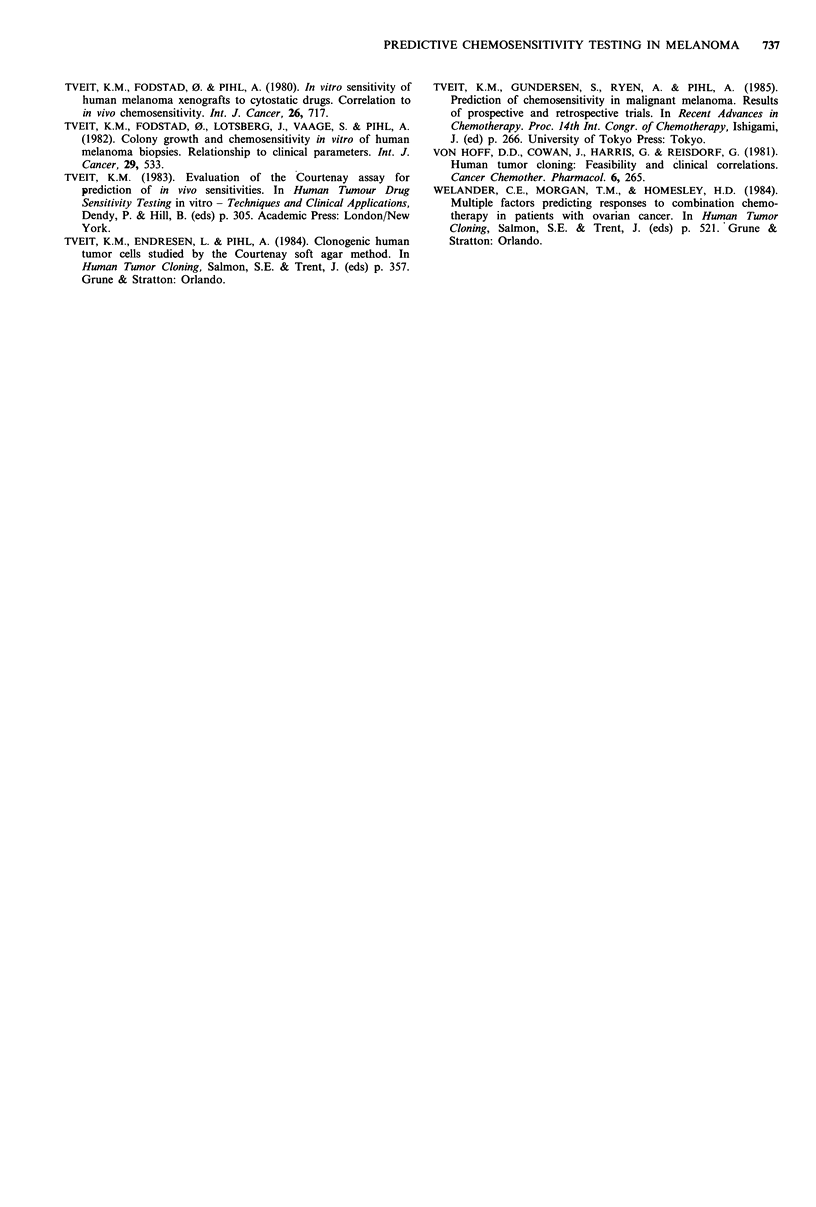# Predictive chemosensitivity testing in malignant melanoma: reliable methodology--ineffective drugs.

**DOI:** 10.1038/bjc.1988.299

**Published:** 1988-12

**Authors:** K. M. Tveit, S. Gundersen, J. Høie, A. Pihl

**Affiliations:** Norwegian Radium Hospital, Montebello, Norway.

## Abstract

A retrospective and a prospective trial were carried out in patients with malignant melanomas to investigate the predictive value of an in vitro chemosensitivity assay based on the Courtenay and Mills soft agar cultivation method. Evaluable in vitro chemosensitivity data for the three agents DTIC, CCNU, and vinblastine were obtained in 153 cases. In the retrospective study in which the patients received chemotherapy without prior knowledge of the test results, 50 in vitro/in vivo correlations (40 patients) were made, and in the prospective study, where patients received the single agent most active in vitro, 55 correlations (45 patients) were performed. In both studies the sensitivity of the test (the ability to identify patients who will respond to chemotherapy) was approximately 100% and the specificity (the ability to identify patients who will not respond) was 87-98%. Depending on whether 'no change' and 'mixed response' were classified as sensitivity or resistance, the predictive value of a negative test was approximately 100% and that of a positive test 37.5-87.5%. The response rate was low in both series, and although it was somewhat higher in the prospective than in the retrospective trial, the difference was not significant. The median survival time was not significantly different in the two treatment series. We conclude that the chemosensitivity assay here used is reliable and has predictive value, but that the chemotherapeutic agents currently available for treatment of melanoma are too ineffective to warrant routine use of the assay in this disease.


Br. J. Cancer (1988), 58, 734–737 © The Macmillan Press Ltd., 1988

### Predictive chemosensitivity testing in malignant melanoma: Reliable methodology – ineffective drugs

K.M. Tveit, S. Gundersen, J. Høie & A. Pihl

*The Norwegian Radium Hospital and Institute for Cancer Research, Montebello, 0310 Oslo 3, Norway*

**Summary** A retrospective and a prospective trial were carried out in patients with malignant melanomas to investigate the predictive value of an *in vitro* chemosensitivity assay based on the Courtenay and Mills soft agar cultivation method. Evaluable *in vitro* chemosensitivity data for the three agents DTIC, CCNU, and vinblastine were obtained in 153 cases. In the retrospective study in which the patients received chemotherapy without prior knowledge of the test results, 50 *in vitro/in vivo* correlations (40 patients) were made, and in the prospective study, where patients received the single agent most active *in vitro,* 55 correlations (45 patients) were performed. In both studies the sensitivity of the test (the ability to identify patients who *will* respond to chemotherapy) was ~100% and the specificity (the ability to identify patients who *will not* respond) was 87–98%. Depending on whether 'no change' and 'mixed response' were classified as sensitivity or resistance, the predictive value of a negative test was ~100% and that of a positive test 37.5–87.5%. The response rate was low in both series, and although it was somewhat higher in the prospective than in the retrospective trial, the difference was not significant. The median survival time was not significantly different in the two treatment series. We conclude that the chemosensitivity assay here used is reliable and has predictive value, but that the chemotherapeutic agents currently available for treatment of melanoma are too ineffective to warrant routine use of the assay in this disease.

During the last decade, starting with the pioneer work of Salmon *et al.*, (1978), a large number of laboratories have attempted to use chemosensitivity testing *in vitro*, based on semi-solid medium, as a guide to the treatment of various human malignant tumours. Several interesting reports on the predictive value of such tests in various human tumours have appeared. Although the initial enthusiasm for predictive testing has declined, such testing may be useful in certain types of cancer (Alberts *et al.*, 1984; Welander *et al.*, 1984; Hanauske *et al.*, 1985) and the prediction concept has prompted valuable basic and clinically relevant studies of cell biology and therapy responses *in vitro*.

Malignant melanomas lend themselves to chemosensitivity testing insofar as they are rather easy to grow in semi-solid medium, and several reports on predictive chemosensitivity testing in this tumour type have appeared. Positive correlations between *in vitro* sensitivity and clinical response have been reported, but a beneficial value of the test systems in the routine chemotherapy of patients has not been established (Meyskens *et al.*, 1981, 1984; Bertelsen *et al.*, 1984; Mackie *et al.*, 1984; Tveit *et al.*, 1982, 1985). Here we report on our experience in a retrospective and prospective trial of malignant melanoma using a cultivation method different from that used by most workers, and also a different calibration method of the *in vitro* results.

### Materials and methods

### *Tumours*

Altogether 402 histologically verified malignant melanomas (metastases or local recurrences), surgically removed from patients admitted to The Norwegian Radium Hospital, were disaggregated by a mechanical procedure employing a stomacher (Tveit *et al.*, 1984). The cell suspension was filtered through a 45 $\mu$M nylon mesh, washed and resuspended in Hams F12 medium supplemented with 15% foetal calf serum, 100 IU ml$^{-1}$ penicillin and 100 $\mu$g ml$^{-1}$ streptomycin. The yield of tumour cells was calculated, and in the cases where a high enough yield was obtained to permit chemosensitivity studies, chemosensitivity experiments were performed. The viability of disaggregated tumour cells, as determined in the phase contrast microscope by scoring intact cells with a bright outline as viable, was in the range 40–80%.

### *Chemosensitivity assay*

In 240 cases, chemosensitivity experiments, including at least three anti-cancer agents, were performed. Cells ($5\times10^5$) were treated with 4 different concentrations of dacarbazine (DTIC, 80, 250, 800 and 2,500 $\mu$g ml$^{-1}$), lomustine (CCNU, 0.04, 0.4, 4 and 40 $\mu$g ml$^{-1}$) and vinblastine (VBL) (0.01, 0.1, 1 and 10 $\mu$g ml$^{-1}$) for 1 h during continuous agitation. In addition, a positive control with abrin (10 $\mu$g ml$^{-1}$) was included. The anti-cancer agents were dissolved in PBS (DTIC, vinblastine and abrin) or cremophor EL (CCNU) and were stored in aliquots at −70°C. DTIC was protected from light both during storage and the experimental procedure. After incubation, the cells were washed twice in PBS and resuspended in complete medium. The number of viable cells was scored, and the soft agar cultures were set up in triplicate.

The soft agar method described by Courtenay and Mills (1978) was used (Tveit *et al.*, 1984). Briefly, cultures of treated and untreated cells were set up in culture tubes by adding 0.2 ml of a suspension of washed and heated rat erythrocytes diluted 1:8, 0.2 ml of the tumour cell suspension (properly diluted to give $2\times10^4$ viable cells in each culture) and 0.6 ml 0.5% agar. After mixing the components and solidification of the agar, the tubes were placed with the caps in the open position in an incubator controlling the exact concentrations of $O_2$ (5%), $CO_2$ (5%) and $N_2$ (90%). On the next day the tubes were sealed, and after ~7 days 1 ml medium was added to each tube. Counting of colonies was usually performed after 2 weeks (alternatively 3 weeks), using a stereo microscope. Colonies of more than 30 cells, or with a diameter of a least 100 $\mu$m were scored. Only experiments with abrin negative controls were included in the study. To evaluate chemosensitivity, at least 30 colonies in the controls were required.

### *Clinical treatment*

In the retrospective trial the patients received single-agent DTIC, CCNU or vinblastine treatment, chosen by the clinician without prior knowledge of the *in vitro* test results on tumour specimens. In the prospective study the clinician

Correspondence: K.M. Tveit.
Received 2 May 1988; and in revised form, 18 July 1988.

waited for the *in vitro* testing and treated the patient with the one of the three agents that was most active *in vitro* (see below). In both studies, DTIC was used at a dosage of $800\,mg\,m^{-2}$, given every 4 weeks, CCNU at a dosage of $120\,mg\,m^{-2}$ given every 6 weeks, and vinblastine at $6\,mg\,m^{-2}$ given every week. Response evaluation was performed after 2 to 3 months by simple measurements of palpable lesions, chest X-rays or CT-scans.

Clinical responses were classified according to international criteria as complete response (CR), partial response (PR) and progressive disease (PD). In addition, mixed response (MR) was defined as more than 50% regression of one or more metastases, while concurrently one or more lesions increased in size or a new one appeared during treatment. No change (NC) was defined as lack of progression of any tumour manifestation for at least 3 months after prior progression.

*Data analysis*

The *in vitro* chemosensitivity was quantitated on the basis of the observed dose-response curves. The $ID_{50}$s (doses required to inhibit colony formation by 50%) were derived from the dose-response curves and correlated with the *in vivo* sensitivity of melanoma xenografts previously measured (Tveit *et al.*, 1980, 1982). By this calibration procedure the *in vitro* chemosensitivity is converted to an *in vivo* sensitivity and is expressed in terms of expected growth delay (EGD). In our earlier studies (Tveit, 1983), we found that an EGD limit of 2.0 was a useful cut-off value for predicting tumours as being either 'sensitive' or 'resistant' *in vivo*.

'Sensitivity' of the test system was defined as the ability of the assay to identify patients who will respond to chemotherapy and 'specificity' as its ability to identify patients who will not respond to chemotherapy. These parameters were expressed as follows:

Sensitivity $= sS/(sS+rS)$, and specificity $= rR/(rR+sR)$, where $s$ and $S$ denote sensitivities *in vitro* and in the clinic, respectively, and $r$ and $R$ resistance *in vitro* and in the clinic, respectively. Moreover, the 'predictive value of a positive test' (PV+) was defined as $sS/(sS+sR)$ and the 'predictive value of a negative test' as $rR/(rR+rS)$.

### Results

Altogether 402 malignant melanomas were cultivated in soft agar and gave evaluable colony formation *in vitro*. Two hundred and eighty-five tumours (71%) formed more than 10 colonies and the colony forming efficiencies obtained ranged from 0.15% up to 20%. Sensitivity testing for DTIC, CCNU and vinblastine was performed in 240 cases, and in 153 cases of these (64%), evaluable chemosensitivity data were obtained for all 3 drugs. In 85 patients, 105 correlations between *in vitro* data and evaluable clinical responses were made.

Figure 1 shows representative dose-response relationships for 10 tumours treated *in vitro* with DTIC, CCNU and vinblastine. It is gratifying that none of the dose-response curves had plateaus. The $ID_{50}$s were derived graphically and converted to EGD values as described in the **Materials and methods** section and in Tveit *et al.*, (1980). In general, the same chemosensitivity patterns were found in metastases compared to local recurrences.

In the retrospective study, 3 partial responses were observed and in addition 4 patients had no change or mixed response. In 43 cases progressive disease was found. In the prospective study, one patient had complete response, 8 had partial response, 6 had no change or mixed response and 40 had progressive disease.

Figure 2 shows correlations between the estimated EGD values and the various categories of clinical responses in the retrospective study (50 cases) and in the prospective study (55 cases). Table I summarizes the results. In the retrospective study, three patients with partial responses all had tumours that were sensitive *in vitro* (EGD<2.0), and of 43 cases with progressive disease, 42 had tumours that were resistant *in vitro* (EGD> =2.0), whereas one had a sensitive tumour. Four cases with mixed responses or no change had sensitive tumours *in vitro*. In the prospective study, 9 patients with partial or complete responses had sensitive tumours *in vitro*. Of 40 cases with progressive disease, 37 had resistant tumours *in vitro*, whereas 3 had sensitive tumours *in vitro*. Three patients with MR or NC had sensitive tumours *in vitro*, whereas 3 other patients had resistant tumours *in vitro*.

The estimation of the 'sensitivity' and 'specificity' of the test and the 'predictive value' of a positive or a negative test was dependent on whether a mixed response and no change were classified as clinically sensitive or as clinically resistant

**Figure 1** Dose-response relationships for 10 melanomas treated *in vitro* with 4 concentrations (see **Materials and methods**) of DTIC, CCNU and vinblastine. Two of the DTIC curves are almost identical and cannot be separated in the graph.

**Figure 2** Correlations between *in vitro* sensitivity (expected growth delay) and clinical responses (CR, PR, MR, NC, PD, see text) in 50 cases, retrospective study (open circles) and 55 cases, prospective study (closed circles).

**Table I** Correlations between *in vitro* sensitivity and clinical response to either DTIC, CCNU or vinblastine in malignant melanomas

| | *Clinical response* | | | |
|---|---|---|---|---|
| *Type of study* | *CR+PR* | *MR+NC* | *PD* | *Number of correlations* |
| *Retrospective* | | | | |
| *In vitro* sensitivity[a] | 3 | 4 | 1 | 50 |
| *In vitro* resistance[b] | 0 | 0 | 42 | |
| *Prospective* | | | | |
| *In vitro* sensitivity[a] | 9 | 3 | 3 | 55 |
| *In vitro* resistance[b] | 0 | 3 | 37 | |

[a]Expected growth delay (EGD) ≧2.0; [b]Expected growth delay (EGD) <2.0.

(Table II). If MR and NC are classified as clinical resistance, in the retrospective study the sensitivity was 100%, the specificity 89%, the predictive value of a positive test (PV+) was 38%, and the predictive value of a negative test (PV−) 100%. If, however, MR and NC are classified as clinical sensitivity, the specificity and PV+ values increase (Table II). In the prospective study, the sensitivity and PV− were 100% if MR and NC are classified as resistance. The specificity was 87% and PV+ was 60%. If MR+NC are classified as sensitive, the consequence is that the sensitivity and PV− values decrease, and the specificity and PV+ values increase. In the prospective study, it turned out that DTIC was most active in 32, CCNU in 14, and vinblastine in 9 of the 55 cases. These findings parallel closely the clinical experience that DTIC is the most active agent in melanomas, more active than CCNU and vinblastine. The results show that relatively more objective clinical responses were obtained in the prospective than in the retrospective study. Thus, PR+CR increased from 6.0% in the retrospective study to 16.4% in the prospective study (not statistically significant, $P=0.09$, Chi-square test), and NC+MR increased from 8.0% to 10.8%. Altogether, 27.2% of the patients in the prospective study had some kind of response, compared to 14.0% in the retrospective study ($P=0.09$).

The median survival was not significantly different in the two trials (7.3 months in the retrospective and 9.1 months in the prospective trial).

### Discussion

Several significant facts emerge from the present study. In the first place, the association between *in vitro* sensitivity and clinical response was highly significant. This shows that the assay here used, which is based on the Courtenay and Mills culture method and calibrated by means of melanoma xenografts, is reliable and has high predictive value. Secondly, the response rate to the agents tested, considered by most workers to be among the most efficacious ones in melanoma, was very low. Although the response rate was higher in the prospective than in the retrospective study, the difference was not statistically significant, and the median survival was not significantly higher in the prospective trial. Thus, our study fails to demonstrate a definite therapeutic value of predictive testing in malignant melanoma.

A serious shortcoming of *in vitro* chemosensitivity testing is the low percentage of evaluable tests (von Hoff *et al.*, 1981). In 240 of the 402 specimens received in the laboratory, a sufficient number of cells was obtained to set up a test with the 3 different agents at 4 different concentrations. Sixty-four per cent of these experiments could be evaluated for chemosensitivity to all agents. This implies that in the majority (62%) of the malignant melanoma specimens received, chemosensitivity data could not be obtained, either due to an insufficient number of cells in the specimen, or to inadequate growth. We do not believe that these figures can be significantly improved as we have made considerable efforts to optimize both the disaggregation and the cultivation procedures (Tveit *et al.*, 1984).

Although the probability of finding a drug capable of inducing a response undoubtedly will increase with increasing number of drugs tested, as shown by Meyskens *et al.* (1981), such extended testing will be possible only in a minority of cases, and we doubt that the increase in effort and expense required will be justified by the results. Thus, in several cases we have performed testing with 6 different agents without finding any of them active (not shown), and we believe that many cases of malignant melanomas may be resistant to all agents currently available.

In spite of the low response rate of melanoma to current chemotherapy, many centres continue to administer chemotherapy routinely to melanoma patients with metastatic disease. A rationale for using a predictive assay in a tumour type such as malignant melanoma, could be to identify the few tumours that are sensitive and to avoid chemotherapy in the majority of patients who have unresponsive tumours. However, in view of the marginal effect of all available chemotherapy regimens with only minor or partial responses of short duration without any prolongation of survival time, we conclude that a routine testing of patients with malignant melanomas is not justified at the present time. This situation may change if more active agents become available. Presently, melanoma patients should, in our opinion, not have chemotherapy with any of the current cytotoxic agents, but should preferably be included in clinical Phase II protocols of promising new agents.

**Table II** Sensitivity, specificity and predictive value of a positive (PV+) or a negative (PV−) test in malignant melanoma, when MR+NC is classified as either clinical resistance (R) or as sensitivity (S)

| | *MR+NC=R* | *MR+NC=S* |
|---|---|---|
| *Retrospective study* | | |
| Sensitivity[a] | 3/3=100% | 7/7=100% |
| Specificity[b] | 42/47= 89% | 42/43= 98% |
| PV+[c] | 3/8= 38% | 7/8= 88% |
| PV−[d] | 42/42=100% | 42/42=100% |
| *Prospective study* | | |
| Sensitivity[a] | 9/9=100% | 12/15= 80% |
| Specificity[b] | 40/46= 87% | 37/40= 93% |
| PV+[c] | 9/15= 60% | 12/15= 80% |
| PV−[d] | 40/40=100% | 37/40= 93% |

[a]$sS/(sS+rS)$; [b]$rR/(rR+sR)$; [c]$sS/(sS+sR)$; [d]$rR(rR+rS)$.

This work was supported by the Norwegian Cancer Society. The skilful technical assistance of Hanne Kleppe Höifödt is gratefully acknowledged.

### References

ALBERTS, D.S., LEIGH, S., SURWIT, E.A., SEROKMAN, R., MOON, T.E. & SALMON, S.E. (1984). Improved survival of patients with relapsing ovarian cancer treated on the basis of drug selection following human tumour clonogenic assay. In *Human Tumor Cloning*, Salmon, S.E. & Trent, J.M. (eds) p.509. Grune & Stratton: Orlando.

BERTELSEN, C.A., SONDAK, V.K., MANN, B.D., KORN, E.L. & KERN, D.H. (1984). Chemosensitivity testing of human solid tumors: A review of 1582 assays with 258 correlations. *Cancer*, **53**, 1240.

COURTENAY, V.D. & MILLS, J. (1978). An *in vitro* colony assay for human tumours grown in immune-suppressed mice and treated *in vivo* with cytotoxic agents. *Br. J. Cancer*, **38**, 77.

HANAUSKE, A.-R., HANAUSKE, U. & VON HOFF, D. (1985). The human tumor cloning assay in cancer research and therapy. *Current Probl. Cancer, Vol. IX*, **12**, 1.

MACKIE, R.M., GAUKROGER, J.M., WILSON, L. & GOLD, J. (1984). Experience with the colony-forming (stem cell) assay of *in vitro* chemosensitivity in the management of patients with advanced malignant melanoma. *Cancer Treat. Rep.*, **68**, 1185.

MEYSKENS, F.L. JR., MOON, T.E., DANA, B. & 6 others (1981). Quantitation of drug sensitivity by human metastatic melanoma colony-forming units. *Br. J. Cancer*, **44**, 787.

MEYSKENS, F.L. JR., LOESCHER, L., MOON, T.E., TAKASUGI, B. & SALMON, S.E. (1984). Relation of *in vitro* colony survival to clinical response in a prospective trial of single-agent chemotherapy of metastatic melanoma. *J. Clin. Oncol.*, **2**, 1223.

SALMON, S.E., HAMBURGER, A.W., SOEHNLEN, B., DURIE, B.G.M., ALBERTS, D.S. & MOON, T.E. (1978). Quantitation of differential sensitivity of human tumor stem cells to anticancer drugs. *N. Engl. J. Med.*, **298**, 1321.

TVEIT, K.M., FODSTAD, Ø. & PIHL, A. (1980). *In vitro* sensitivity of human melanoma xenografts to cytostatic drugs. Correlation to *in vivo* chemosensitivity. *Int. J. Cancer,* **26,** 717.

TVEIT, K.M., FODSTAD, Ø., LOTSBERG, J., VAAGE, S. & PIHL, A. (1982). Colony growth and chemosensitivity *in vitro* of human melanoma biopsies. Relationship to clinical parameters. *Int. J. Cancer,* **29,** 533.

TVEIT, K.M. (1983). Evaluation of the Courtenay assay for prediction of *in vivo* sensitivities. In *Human Tumour Drug Sensitivity Testing* in vitro – *Techniques and Clinical Applications,* Dendy, P. & Hill, B. (eds) p. 305. Academic Press: London/New York.

TVEIT, K.M., ENDRESEN, L. & PIHL, A. (1984). Clonogenic human tumor cells studied by the Courtenay soft agar method. In *Human Tumor Cloning,* Salmon, S.E. & Trent, J. (eds) p. 357. Grune & Stratton: Orlando.

TVEIT, K.M., GUNDERSEN, S., RYEN, A. & PIHL, A. (1985). Prediction of chemosensitivity in malignant melanoma. Results of prospective and retrospective trials. In *Recent Advances in Chemotherapy. Proc. 14th Int. Congr. of Chemotherapy,* Ishigami, J. (ed) p. 266. University of Tokyo Press: Tokyo.

VON HOFF, D.D., COWAN, J., HARRIS, G. & REISDORF, G. (1981). Human tumor cloning: Feasibility and clinical correlations. *Cancer Chemother. Pharmacol.* **6,** 265.

WELANDER, C.E., MORGAN, T.M., & HOMESLEY, H.D. (1984). Multiple factors predicting responses to combination chemotherapy in patients with ovarian cancer. In *Human Tumor Cloning,* Salmon, S.E. & Trent, J. (eds) p. 521. Grune & Stratton: Orlando.